# The Ecological Coherence of Temperature and Salinity Tolerance Interaction and Pigmentation in a Non-marine *Vibrio* Isolated from Salar de Atacama

**DOI:** 10.3389/fmicb.2016.01943

**Published:** 2016-12-01

**Authors:** Karem Gallardo, Jonathan E. Candia, Francisco Remonsellez, Lorena V. Escudero, Cecilia S. Demergasso

**Affiliations:** ^1^Centro de Biotecnología, Universidad Católica del NorteAntofagasta, Chile; ^2^Departamento de Ingeniería Química, Universidad Católica del NorteAntofagasta, Chile; ^3^Centro de Investigación Científico Tecnológico para la MineríaAntofagasta, Chile

**Keywords:** *Vibrio*, osmotic-stress, halotolerant, psychrotolerant, prodigiosin

## Abstract

The occurrence of microorganisms from the *Vibrio* genus in saline lakes from northern Chile had been evidenced using Numerical Taxonomy decades before and, more recently, by phylogenetic analyses of environmental samples and isolates. Most of the knowledge about this genus came from marine isolates and showed temperature and salinity to be integral agents in shaping the niche of the *Vibrio* populations. The stress tolerance phenotypes of *Vibrio* sp. Teb5a1 isolated from Salar de Atacama was investigated. It was able to grow without NaCl and tolerated up to 100 g/L of the salt. Furthermore, it grew between 17° and 49°C (optimum 30°C) in the absence of NaCl, and the range was expanded into cold temperature (4–49°C) in the presence of the salt. Other additional adaptive strategies were observed in response to the osmotic stress: pigment production, identified as the known antibacterial prodigiosin, swimming and swarming motility and synthesis of a polar flagellum. It is possible to infer that environmental congruence might explain the cellular phenotypes observed in *Vibrio* sp. considering that coupling between temperature and salinity tolerance, the production of antibacterial agents at higher temperatures, flagellation and motility increase the chance of *Vibrio* sp. to survive in salty environments with high daily temperature swings and UV radiation.

## Introduction

Extremophiles are considered microorganisms that require, for optimal growth, conditions that are not conducive to human life, like low or high pH, extreme temperatures, chemical oxidizing agents, hypersalinity or certain types of ultraviolet light (Sandle and Skinner, 2013). Among extremophiles, there are some microorganisms that are known as salt-tolerating (halotolerant) because they are able to grow in presence of relatively high salt concentrations and in absence of salt; and others are known as salt-loving (halophilic) because halophiles require a saline environment for growth (Ventosa et al., 1998; Oren, 2013).

In previous works, some halophilic microorganisms from the *Vibrionaceae* family have been studied, like *Photobacterium*, *Listonella*, *Moritella*, *Salinivibrio*, and *Vibrio* genus (Kamekura and Kushner, 1984; Garcia et al., 1987; Shieh, 2003; Borić et al., 2011; Lucena et al., 2012). They are facultative anaerobic, Gram-negative, and rod-shaped bacteria; these organisms are ever-present in estuarine, coastal, oceanic water, and marine sediments (Shieh, 2003; Borić et al., 2011; Lucena et al., 2012). Few studies have been reported about microorganisms isolated from Chilean salt lakes (Campos, 1993; Monteoliva-Sánchez et al., 2002; Demergasso et al., 2004).

*Salinivibrio costicola* and *Vibrio ruber* are the most studied halophilic eubacteria. They can grow in a salinity range from 0.5 to 12% and from 0.05 to 17% of salinity, respectively, and in a range of temperature from 5 to 45 and from 10 to 44°C, respectively (Supplementary Table 1). The adaptation strategies developed by those microorganisms to survive environmental extremes, and perturbations of salinity and temperature have been extensively studied and can be summarized as follows: salinity, (i) balance their cytoplasm with the osmotic pressure of the external medium by accumulation of osmoprotectants (like sugars, polyols, amino acids, and their derivatives) either by uptake from the environment or by their *de novo* synthesis (Ventosa et al., 1998; Danevcic et al., 2005; Zhu et al., 2008; Danevcic, 2014); (ii) modification of the lipid composition by changing of both polar head groups and acyl chains, the amount of unsaturated fatty acids and the occurrence of hydroxyl fatty acids (Hanna, 1984; Adams and Russell, 1992; Ventosa et al., 1998; Danevcic, 2014); (iii) changes in the membrane reaction potential of *V. ruber* (Danevcic, 2014); (iv) activity of ion pumps in *S. costicola* membranes represented by two alternative mechanisms, a Na^+^/H^+^ antiport and the presence of a primary respiration-driven Na^+^ pump (Ventosa et al., 1998; Müller and Oren, 2003); (v) changes in the composition and activity of ribosomal proteins, on the protein turnover, in the composition and activity of glycolytic and electron transport active proteins involved in central metabolic pathways, like pyruvate kinase and dehydrogenase (de Médicis and Rossignol, 1979; Ventosa et al., 1998; Danevčič and Stopar, 2011); (vi) increase of the secondary metabolite production – prodigiosin- by *V. ruber* with known capacity as a transmembrane chloride anion carrier among other properties (Danevcic, 2014; Seganish and Davis, 2005); and temperature, (vii) changes in the prodigiosin production by *V. ruber* with an optimum at 28°C (Danevcic, 2014); (viii) change in the polar lipid head groups composition, in saturation of the phospholipid fatty acyl composition and in the mean acyl chain length of *S. costicola* (Adams and Russell, 1992). The ecophysiological response to other relevant environmental factors which determines the growth and survival of those microorganisms like nutrients availability, viscosity, and UV radiation have been also reported (Danevcic, 2014).

According to some authors, *Vibrio* presents a red pigment known as prodigiosin (representative of the prodiginines family), which is a secondary metabolite that was first characterized from *Serratia marcescens* and it was later found to be produced by other bacteria, mostly members of the proteobacteria (Suryawanshi et al., 2015). Several bacteria that produce prodigiosin were characterized as inhabiting open habitats with high microbial diversity, and therefore there was intense competition between the community members (Suryawanshi et al., 2015). In addition, some environmental factors can influence prodigiosin production, like temperature, pH, media composition, and salinity, among others (Venil, 2009). For instance, one of the major requirements for effective pigment production and for the growth of isolated marine *Vibrio* sp. is NaCl (Kirishna et al., 2014). Moreover, some other research groups reported that NaCl is required for growth and pigmentation in *Serratia marcescens* (Silverman and Munoz, 1973; Allen et al., 1983). Besides the impact of ion concentrations, the effect of the temperature on the activation of enzymes involved in prodigiosin biosynthetic pathway (Williams et al., 1971; Casullo de Araújo et al., 2010; Starič et al., 2010) has also been reported. However, most of the studies were related to room temperature or optimal temperature.

Protective or metabolic roles, among others, have been attributed to prodigiosin; some of these roles are related to energy spilling reaction, air dispersal of bacteria, light storage (energy), UV survival, anion exchange and antimicrobial activity (Burger and Bennett, 1985; Ryazantseva et al., 1995; Seganish and Davis, 2005; Haddix et al., 2008; Venil, 2009; Starič et al., 2010; Borić et al., 2011; Danevcic, 2014). Besides, algicidal activity was also reported and proposed to be used in the control of bloom-forming red-tide phytoplanktons (Kim et al., 2008). Furthermore, prodigiosin 1, parent of compounds isolated from *Serratia marcescens* as described by Fürstner (2003), transports chloride ions into the cell across phospholipid vesicles by functioning as H^+^/Cl^-^ symporters. Then, this pigment operates as chloride anion carrier, but it is also able to exchange chloride for nitrate anions without any change in the internal pH during transmembrane transport by functioning as antiporter (Seganish and Davis, 2005; Díaz et al., 2007).

In addition, some biotechnological roles of prodigiosin were already reported to have immunosuppressive, antiproliferative, antimalarial, bactericidal, UV protection, antioxidant, and antitumor properties (Burger and Bennett, 1985; Venil, 2009; Borić et al., 2012; Arivizhivendhan et al., 2015; Darshan and Manonmani, 2015; Lapenda et al., 2015).

An isolated strain of *Vibrio* sp. (Supplementary Figure 1) from Salar de Atacama (Supplementary Table 2) showed an ample range of saline as well as temperature tolerance. Both salinity and temperature affected morphology (flagellation and aggregation), growth, motility and pigment production (prodigiosin). The cellular phenotypes observed were described and the ecological significance was discussed.

## Materials and Methods

### Strains and Culture Conditions

All chemical used in these experiments were purchased from Merck and Difco.

A strain of *Vibrio* genus (*Vibrio* sp. Teb5a1) isolated from Laguna de Tebenquiche in Salar de Atacama was used to work during these experiments. Initially, the microorganism *Vibrio* sp. was grown at 30°C in an orbital shaker at 100 rpm in a seawater medium (salinity 3% approximately), with yeast extract and peptone. The isolations were achieved in plates with the same medium and bacteriologic agar. Subsequently, modifications of this medium were performed to control the growing conditions (“defined medium”). This new medium was prepared according to (Shieh, 2003); it comprised peptone (6 g/L), yeast extract (2 g/L), MgSO_4_x7H_2_O (3 g/L), CaCl_2_ (0.01 g/L), KCl (0.6 g/L) and variable NaCl concentrations. Incubation conditions were 30°C in an orbital shaker (100 rpm). Additionally, *Vibrio* sp. was isolated in plates with the same defined medium and bacteriologic agar.

### Immediate Adaptation of *Vibrio* sp. to Salt

To perform the immediate adaptation assays, the microorganism was grown in the defined medium without NaCl until its exponential phase was reached at 30°C and 100 rpm orbital shaking. Successively, this culture was used to inoculate media with different NaCl concentrations (from 0 to 150 g/L NaCl) and incubated at the same temperature (30°C). Three biological replicates were used to perform the adaptation of *Vibrio* sp.

### Identification of Optimum Growth Conditions for *Vibrio* sp.

The defined medium was used to identify the optimum conditions for *Vibrio* sp. growing, as described previously. The growth experiments were performed to identify the optimum temperature and the optimum NaCl concentration, which were achieved in a range from 4 to 49°C and from 0 to 150 g/L of NaCl, respectively. The growth of each culture was checked using a Neubauer camera of 0.01 mm depth (Labolan) in an optical microscope (Olympus). Additionally, the growth of *Vibrio* sp. was checked by optical density at 600 nm wavelength in a UV-visible spectrophotometer (Perkin Elmer). Three biological replicates were used to identify the optimum growth conditions (temperature and salinity) for *Vibrio* sp.

### Extraction of Red Pigment in *Vibrio* sp.

Considering that *Vibrio* sp. produced a red pigment, three methods (Cang et al., 2000; Song et al., 2006; Alihosseini et al., 2008) for pigment extraction with minor modifications were used to identify the pigment by UV/Vis absorption and to compare it with published results. Method 1 (Song et al., 2006): 25 mL of supernatant from cultures of *Vibrio* sp. with 25 mL of 95% Ethanol pH 3 (Merck) were mixed in a separatory funnel during 30 s. After 15 min, 25 mL of chloroform (Merck) were added to the previous solution and mixed again for 30 s. The lower phase (polar phase) was stored at room temperature in a dark flask until it was used again. Method 2 (Cang et al., 2000): 25 mL of supernatant from *Vibrio* sp. cultures with 25 mL of Acetone (Merck) were mixed in a separatory funnel during 30 s. After 10 min, 25 mL of chloroform pH 3 (Merck) were added and mixed. Again, the polar phase was stored at room temperature in darkness until it was used. Method 3 (Alihosseini et al., 2008): 25 mL of supernatant from *Vibrio* sp. cultures with 25 mL of chloroform (Merck) were mixed in a separatory funnel during 30 s. After 1 min, the polar phase was collected and stored at room temperature in a dark flask. In addition, absorption of UV/Visible light (UV/Vis Lambda EZ 301 spectrometer, PerkinElmer) of all tree collected samples (from methods 1, 2, and 3) was performed, considering as blank chloroform.

### Purification of Extracted Red Pigment from *Vibrio* sp.

The cellular pellet from 6 L of *Vibrio* sp. was obtained by centrifugation (Centrifuge Eppendorf 5804R) during 10 min at 9000 rpm. This pellet was dried at 80°C for 18 h in a sterile petri dish. Two chromatographic columns were prepared: “first column”, 5 g of dried pellet of *Vibrio* sp. plus 10 g of silica gel were loaded into 100 g of pure silica gel column chromatography (Silica gel 60, 0.063–0.200 mm, Merck). Samples collected from this first column were analyzed by UV/vis absorbance spectroscopy. “Second column”, 389 mg of purified (and dried) pellet from the first column plus 5 g of silica gel were loaded into 60 g of pure silica gel column chromatography (Silica gel 60, 0.063–0.200 mm, Merck). Samples collected from this second column were used for mass spectrometry analysis. To remove air bubbles, 1 L of *n*-hexane were loaded into both columns. Polarity of solvents were changed as follow: 10:0, 9:1, 7:3, 6:4, 1:1, 4:6, 3:7, 2:8, 1:9 *n*-hexane:chloroform. Along with polarity changes, fractions were collected and an aliquot was loaded in a 5 cm × 5 cm silica gel TLC plate (Silica gel 60 F254, Merck) to check the isolation of new spots. The elution of fractions in the TLC plates was done with *n*-hexane:Ethyl acetate in a proportion 95:5. Once different compounds were appearing, the polarity was increased. Visualization of spots was done under UV/Vis light, and five methods were used as Supplementary Table 3 shows.

### Identification of Red Pigment by Mass Spectrometry (MS)

In order to determine whether red pigment corresponds to the prodiginine family members, samples were analyzed by mass spectrometry. Fractions from the second column (as it was mentioned before) were analyzed by Mass spectrometry through an HPLC system Agilent 1100 (Agilent Technologies Inc., Folsom, CA, USA) online coupled to ESI-ITMS (Esquire 4000) at the mass spectrometry facility of Universidad de Chile. Separation of the sample was performed through a C18 column (Luna 150 mm × 4.6 mm, 5 μm, 100 Å; Phenomenex, Inc.) during 60 min gradient from 0 to 98% buffer B (details are shown in Supplementary Table 4). Ionization was at 325°C, 30 psi and with nitrogen as the gas carrier. Data analysis was performed with Data Analysis 3.2 (Bruker Daltonik GmbH, Germany).

### Effect of NaCl Concentration in the Motility of *Vibrio* sp.

Two types of motility assays were done (Roeßler et al., 2000) to observe the swarming and swimming motility. For swarming phenomenon, the microorganism was grown in the solidly defined medium with 0.5% of bacteriologic agar and the cultures were inoculated into the agar. For swimming phenomenon, *Vibrio* sp. was grown in the solidly defined medium with 0.3% of bacteriologic agar and the cultures were inoculated with a drop over the agars. Both assays were done in the absence (0 g/L NaCl) and in the presence of NaCl (25 and 100 g/L NaCl), as well as at low temperature (17°C). Three biological replicates were used to study the effect of salinity in the motility of *Vibrio* sp.

### Transmission Electron Microscopy (TEM)

The morphology and the presence of flagellum, in presence and absence of NaCl, were analyzed as previously reported (Gonzalez and Jensen, 1998). The cells were grown until its lag phase with 0, 25, 50, and 100 g/L of NaCl; then cells were washed with ultra-pure water. Later, the cells were resuspended to 0.5 of optical density at A_600_, and aliquots of the cellular suspensions of 10 μL were taken and air-dried on coated grids. Finally, cells were examined under a transmission electron microscope (Philips Tecnai 12) operating in the scanning transmission mode at 80 kV (Pontificia Universidad Católica de Chile).

### Effect of Prodigiosin in the DNA Cleavage

DNA cleavage experiments were performed with 0.4 μg plasmid DNA obtained from an isolate from the salt flat environment using the QIAprep Spin Miniprep (QIAGEN) and the QIAquick PCR purification kits for extraction and purification, respectively. DNA was quantified using a NanoDrop UV-Vis Spectrophotometer.

The plasmid DNA was incubated, either in the absence or presence of 4% v/v of the purified extract and 120 μM Cu II, in 10 mM 3-(*N*-Morpholino)-propanesulfonic Acid-Acetate buffer (MOPS buffer) (pH 7.4), 75 M NaCl, 10% v/v Acetonitrile at 37°C for 0, 30, 60, 90, and 120 min. After incubation, DNA samples were run on horizontal agarose gels (0.8%) containing ethidium bromide (0.2 μg/mL) in 0.5 Tris-acetate-EDTA (1x TAE) buffer for 1 h at 85 V.

## Results

### Effect of Salt Concentration on Growth of *Vibrio* sp.

**FIGURE 1:**
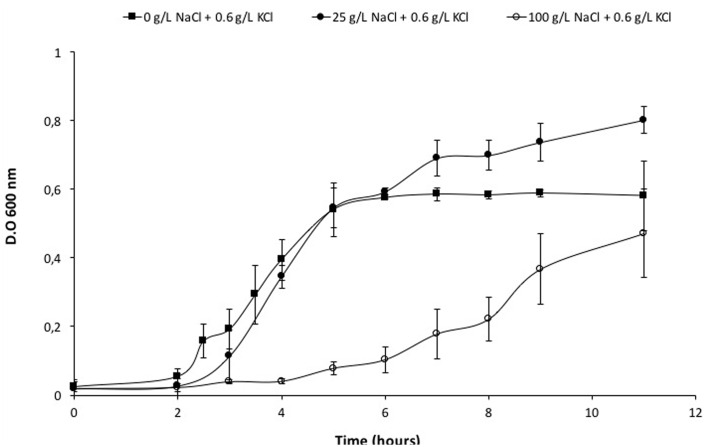
**Adaptation growth curves of *Vibrio* sp. in presence of NaCl.** *Vibrio* sp. cells were adapted previously to grow in presence of 0 g/L NaCl and KCl (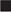), 25 g/L NaCl (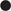) and 100 g/L NaCl (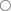). New cultures were prepared from these cells and used to perform their respective curves.

Initially, *Vibrio* sp. was grown in a saline medium (seawater). To control the growth conditions, the microorganism was grown successfully in a defined medium containing 25 g/L of NaCl, and the same characteristics (pigment generation and short duplication time) were observed. Afterward, *Vibrio* sp. was adapted to grow in the absence of NaCl as shown in **Figure 1** and Supplementary Figure 2. In absence of salt and in a medium with up to 25 g/L NaCl, the culture grew immediately; besides, at 50 g/L of NaCl, a longer lag phase evidenced a slower adaptation. Experiments with higher salt concentration (150 g/L NaCl) did not yield positive growth results (data not shown). When the microorganism was already adapted, the optimum conditions of growth were identified. For instance, *Vibrio* sp. was able to grow in absence of NaCl, with a generation time less than 2 h and could tolerate up to 100 g/L NaCl, with a generation time five folds longer than in the absence of salt (Supplementary Figure 3; Supplementary Table 5B).

### Effect of Temperature on Growth of *Vibrio* sp.

**FIGURE 2:**
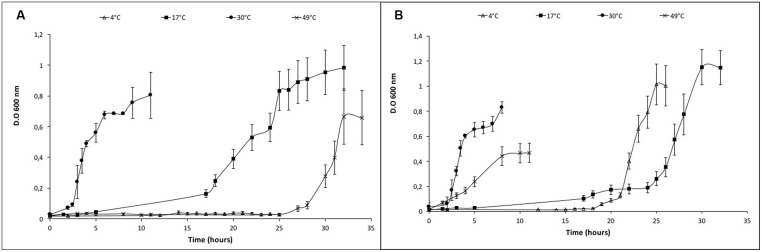
**Growth curves of *Vibrio* sp. at different temperatures in presence and absence of NaCl. (A)** *Vibrio* sp. cells grown in absence of salt (0 g/L NaCl) **(B)** *Vibrio* sp. cells grown in presence of salt (25 g/L NaCl). The temperature was changed from 4° to 49°C under both growth conditions.

After the identification of the optimum salt concentration for growing at 30°C, the microorganism was grown in a range of temperature (from 4 to 49°C) in the absence of NaCl and with 25 g/L NaCl. In the absence of salt, it was able to grow between 17 and 49°C with different lag phases (**Figure 2A**; Supplementary Figure 4A). The range of temperature for growth was extended when *Vibrio* sp. grew with 25 g/L NaCl from 4 (with a generation time of 2, as shown in Supplementary Table 5A) to 49°C. In addition, the lag phases at temperatures below and above the optimum were reduced (**Figure 2B**; Supplementary Figure 4B). The maximal accumulation of the red pigment was evidenced during the growth of *Vibrio* sp. between 26 and 40°C when NaCl was amended. It was reduced below 17°C and there was no pigmentation at 4°C (Supplementary Figure 5).

### Extraction, Purification, and Identification of Red Pigment in *Vibrio* sp.

Solvent extraction of the red pigment through the previously reported methods (Cang et al., 2000; Song et al., 2006; Alihosseini et al., 2008) showed that the maximal absorbance for methods 1 and 3 was 539 nm, whereas, for method 2, the maximal absorbance was 534 nm wavelength (Supplementary Figure 6). Both values for absorbance correlate with prodigiosin or prodigiosin-like compounds (Gerber, 1969).

Purification of the red pigment using the “first column” showed three different compounds related to Prodiginines. According to TLC plates, collected fractions from 336 to 394 correspond to the first compound mixtures, collected fractions from 395 to 423 correspond to the second compound mixtures and collected fractions from 424 to 487 correspond to the third compound mixtures (Supplementary Figure 7). All these fractions were collected in gradients 7:3 (for first and second compound mixtures) and 6:4 (for third compound mixtures) *n*-hexane:Ethyl acetate, respectively. UV/Vis spectroscopy confirmed the presence of three different compounds, and the maximal absorbance was observed at 535, 538, and 533 nm (Supplementary Figure 8). Absorbance below 400 nm corresponds to different compounds or contamination, which were contained in the previous and later prodiginine-containing fractions (as shown in Supplementary Figure 8).

Identification of prodiginines by LC-MS/MS confirmed the presence of more than fifteenth different compounds. Among these compounds, undecylprodiginine, methyldodecylprodiginine, cycloprodigiosin, and prodigiosin were identified, which are the most often reported (Supplementary Table 6). The identification was done comparing the observed fragmentation spectra (in our samples) with those reported by several authors, as it is cited in Supplementary Table 6. However, there were some fragmentation spectra that could not be assigned because there are not reports about that. Therefore, to verify if these MS/MS spectra represent new compounds, additional experiments, like ^13^C NMR and ^1^H NMR, would be necessary.

### Effect of Salinity in Prodigiosin Production of *Vibrio* sp.

**FIGURE 3:**
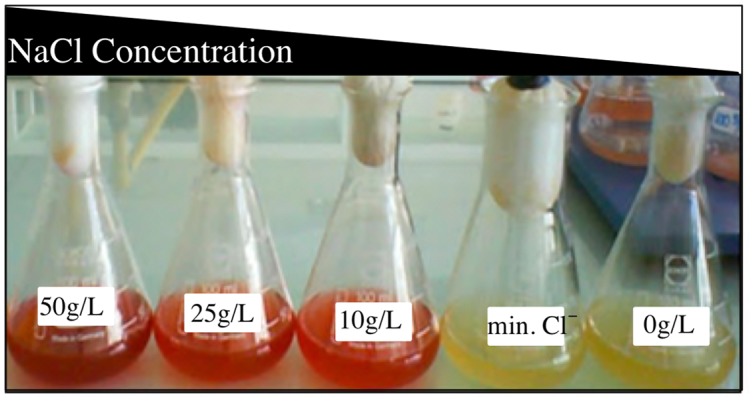
**Effect of salinity on the pigmentation of *Vibrio* sp.** Cells were previously adapted to grow in presence of different salt concentration and temperatures, showing non-accumulation of pigment in absence of NaCl.

It was observed the production of a salt-dependent pigment in each experiment. This pigment is responsible for the red color and it is known as prodiginines. The pigment production showed a direct effect of the salt concentration of the growth medium (**Figure 3**). This pigment production was increased when the NaCl concentration reached up to 100 g/L of NaCl. Assays with higher salt concentration (medium with up to 150 g/L of NaCl) were done but the microorganism did not grow (data not shown).

### Effect of Salinity on Motility of *Vibrio* sp.

**FIGURE 4:**
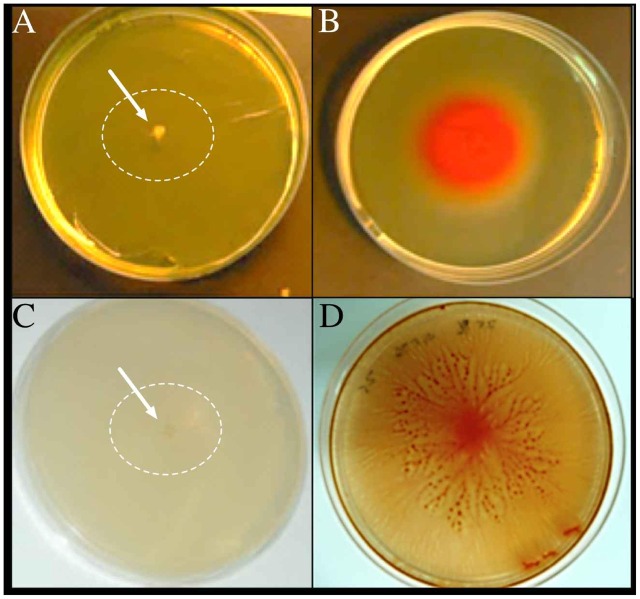
**Swimming and swarming motilities of *Vibrio* sp.** The microorganism was adapted to grow with 0 g/L NaCl **(A,C)** and 25 g/L NaCl **(B,D)**. Cells were inoculated in their respective salts concentration in the swim **(A,B)** and swarm **(C,D)** plates and photographed after 72 h of incubation at 30°C. The arrows and white circles highlight the single colonies in absence of NaCl.

**FIGURE 5:**
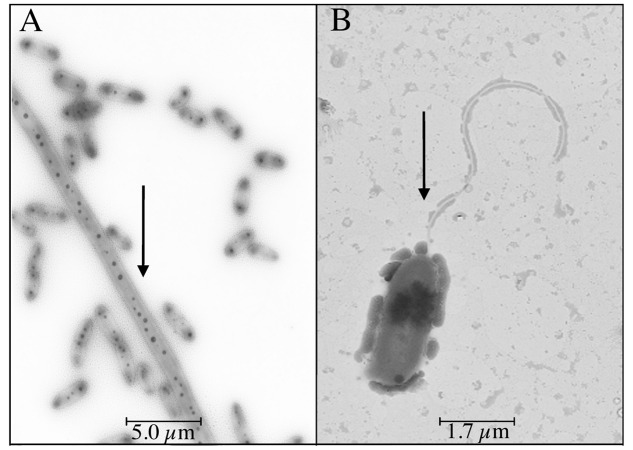
**NaCl-dependent flagellum in *Vibrio* sp.** Cells were grown in 0 g/L **(A)** and 25 g/L **(B)** NaCl and analyzed by transmission electron microscopy. Arrows indicate the presence of dense granules and flagella, respectively.

*Vibrio* sp. showed swimming and swarming motility, which were salt concentration dependent, as shown in **Figure 4**. Both swimming and swarming motility were observed in cultures amended with NaCl from 0 to 100 g/L NaCl; however, the highest motility radio was observed at 25 g/L of NaCl. Motility at higher NaCl concentration (150 g/L of NaCl) was not seen and a filament structure was detected (data not shown). Finally, TEM image revealed that *Vibrio* sp. cells grown in the presence of NaCl presented a single polar flagellum, and cells grown in absence of NaCl evidenced the presence of dense granules, presumably polyphosphate granules (Seufferheld et al., 2008) (**Figure 5**; Supplementary Figure 9). In addition, preliminary information suggested that motility and prodigiosin production might be specifically chloride-dependent (Supplementary Table 7).

### Effect of Prodigiosin in the DNA Cleavage

As can be seen from the agarose gel depicted in Supplementary Figure 10, neither the pigment extract *per se* (lane 6) nor Cu II alone (lane 7) damage purified double-stranded plasmid DNA of the isolate (lane 3). In contrast, a combination of both is very effective. The progress of strand cleavage caused by the complex with increasing incubation time is depicted in lanes 8 ± 12. It is clearly visible that the most relaxed form, which is not evidenced in the controls, constantly gains intensity at the expense of the supercoiled form (band 5).

## Discussion

The characterization of the physiology and morphology changes of *Vibrio* sp. isolated from Salar de Atacama are reported, especially physiological and morphological changes induced by osmotic stress. Very little is known about the presence of microorganisms from the genus *Vibrio* in Chilean salt lakes (Prado et al., 1991). For instance, Prado et al. (1991) isolated 161 moderately halophilic Gram-negative bacteria (including *V. costicola*). Our microorganism *Vibrio* sp. has 97% 16S rRNA similarity with *V. ruber* DSM 14379 (Danevcic, 2014) and 98% with *V. ruber* VR1 (Shieh, 2003). Supplementary Figure 1 shows the phylogeny of *Vibrio* sp. (Teb5a1 strain) and suggest that it is a novel species of the genus *Vibrio* with 97–98% of 16S rRNA gene sequence similarity with the other members. All three (*Vibrio* sp. Teb5a1, *V. ruber* DSM 14379 and *V. rube*r VR1) are able to grow in a broad range of NaCl concentrations. However, *Vibrio* sp. (Teb5a1 strain) can also grow in the absence of NaCl; as a consequence, it must be characterized as halotolerant. *Vibrio* sp. (Teb5a1 strain) was also adapted and able to grow in a wider range of temperature (Supplementary Table 1; Supplementary Figure 4; **Figure 2**), additionally indicating that it is a psychrotolerant strain.

Furthermore, a relationship between temperature and salinity stress parameters (**Figure 2**; Supplementary Figure 4) was observed, resembling the relationship that has been described in several marine bacterial species within the genus *Vibrio* and *Salinivibrio* (Adams and Russell, 1992; Ventosa et al., 1998). This resemblance could explain a linking between physiological, ecological and evolutionary aspects in determining their niche shapes (Materna et al., 2012). Moreover, the coupling between temperature and salinity tolerance might be explained by the effect of chaotropic ions (like chloride) that may counter the macromolecular rigidification induced by low temperature (Chin et al., 2010). Then, it can be assumed that the effect produced by chaotropic agents (like chloride) increases the chance of *Vibrio* sp. to survive in salty environments with high daily temperature swings (Supplementary Table 5). In addition, the relationship between optimum salt concentration and growth temperature in *S. costicola* has been proposed to be due to their effects on membrane lipid phase stability at temperatures below the optimum (Adams and Russell, 1992).

Moreover, a red color pigment was observed in *Vibrio* sp. as the result of prodiginine (prodigiosin) accumulation when the medium was amended with 25 g/L of NaCl (**Figure 3**). Purification and characterization of the red pigment by LC-MS/MS confirmed that it corresponds to prodigiosin and prodigiosin-like compounds (more than 15 different species were identified, as shown in Supplementary Table 6). Unlike to our strain *Vibrio* sp., fewer compounds of prodigiosin or its derivatives have been identified in a single microorganism in previous reports. For instance, Kim et al. (2008) reported in three main constituents in *Hahella chejuensis* that were identified by LC-MS/MS: prodigiosin, norprodigiosin, and undecylprodiginine. Besides, they also reported four prodigiosin analogs but in small quantities: 2-methyl-3-propyl-prodiginine, 2-methyl-3-butyl-prodiginine, 2-methyl-3-hexyl-prodiginine, and 2-methyl-3-heptyl-prodiginine (Kim et al., 2008). Tsao et al. (1985) identified some derivatives of prodigiosin by UV, MS, and HNMR in *Streptomyces coelicolor A3(2)*, like undecylprodigiosin, butylcycloheptylprodiginine, and metacycloprodigiosin.

Additionally, prodiginine accumulation in *Vibrio* sp. showed temperature dependency, being the optimum temperature 25°C. However, the ideal temperature for the microbial production of this particular pigment cannot be generalized. For instance, Starič et al. (2010) reported that *V. ruber* DSM 14379 did not produce pigmentation at low (<15°C) and high temperatures (>43°C). Casullo de Araújo et al. (2010) reported that high temperatures (over 30°C) affected the prodigiosin production in *S. marcescens UCP 1549*, and the ideal temperature for pigment production was 28°C. They reported that the activity of one or more enzymes involved in prodigiosin synthesis is affected at high temperatures (Casullo de Araújo et al., 2010). Williams et al. (1971) defined 27°C as the optimum temperature for prodigiosin production in *S. marcescens*, and that the pigment is synthesized during the stationary phase of growth. Further, they determined protein production and reported that, when bacteria were incubated at 38°C, prodigiosin was not produced, and fewer proteins were formed during the incubation period (Williams et al., 1971). Thus, similar to these previous reports, the prodigiosin biosynthesis in *Vibrio* sp. is affected by high or low temperatures. At 17°C and over 40°C the pigmentation production is already affected. However, in this study, it was not determined which enzyme or protein involved in the biosynthetic pathway is inactivated or perhaps degraded at different growth conditions. Five proteins comprise the prodigiosin biosynthetic pathway: PigA, G, H, I, and J (Garneau-Tsodikova et al., 2006). Quantification of these proteins at different growth temperatures would give a notion of the changes produced by temperature in the prodigiosin synthesis.

Furthermore, preliminary results suggest that the prodigiosin production might be specifically chloride-dependent in *Vibrio* sp. (Supplementary Table 7). Strict chloride dependence of growth or significant stimulation was first reported for *H. halophilus* (Claus et al., 1983; Roeßler and Muller, 1998) and then it has also been described in other microorganisms including *V. fischeri* (Roeßler et al., 2003), at high salt concentrations. Interestingly, germination of endospores, activation of compatible solute transporters, flagella production and motility are among the specific physiological processes described as chloride-dependent (Dohrmann and Muller, 1999; Roeßler et al., 2000; Averhoff and Müller, 2010). Further, Roeßler and Muller (2002) found that chloride ions stimulate *fliC* gene expression, which encodes the major subunit of the flagellum, and regulates the protein FliC synthesis in *H. halophilus*. In addition, the accumulation of both chloride and compatible solutes observed in the moderately halophilic chloride-dependent *H. halophilus* to cope with elevated salinities has been proposed as a hybrid strategy that would represent an intermediate step in the evolution of salt adaptation (Saum et al., 2013). Those reports reinforce the suggestion that *Vibrio* sp. grown only in NaCl-based medium (not in NaBr or Na_2_SO_4_) produces the pigment as has been preliminary observed (Supplementary Table 7).

Besides, the morphology of *Vibrio* sp. was also affected by NaCl concentration (**Figure 5**). Flagellation dependent on NaCl has been previously reported by Rubiano-Labrador et al. (2014, 2015), and Flagellin protein (among others) was identified by mass spectrometry in *Tislia consotensis* grown in 4% of NaCl, but it was not observed at 0.5% NaCl. This protein is the evidence of bacterial flagella and it forms helical chains around the hollow core of the flagellar filament (Rubiano-Labrador et al., 2014, 2015).

In addition, the motility of *Vibrio* sp. was also affected by the presence of NaCl (**Figure 4**) and, perhaps, specifically by chloride ions (data not shown). Halang et al. (2013) reported that in *V. cholerae* chloride ions act as chaotropic agents and enhance cell motility. For instance, the nano-machine flagellum is composed of several subunits, MotA and MotB in H^+^-dependent, or PomA and PomB in Na^+^-dependent flagella (Halang et al., 2013). Chloride ions disrupt hydrogen bonds between water molecules that interact with PomB protein, specifically with D23 and S26 residues. This disruption facilitates the access of sodium ions to PomA and PomB proteins and, then, the flagellum can rotate and the bacteria present motility (Halang et al., 2013). Furthermore, it has been reported that the *Vibrio* motility greatly influences the infectivity of *V. cholera* (Butler and Camilli, 2004) and, on the contrary, reduced motility is induced by starvation in *V. vulnificus* (Chen and Chen, 2014). In both cases, the change means a competitive advantage.

The preliminary confirmation of the ability of the pigment produced by *Vibrio* sp. to damage double-stranded DNA in the presence of Cu II cations has allowed us to infer the ecological significance of this adaptation. The oxidative cleavage of dsDNA to ssDNA by prodigiosin in presence of copper has been reported before (Melvin et al., 2000, 2002). The exact mechanism of this oxidative cleavage is still not clear, but it is known that the cytotoxic activity is mediated by oxidation of the electron-rich polypyrrole molecule (Melvin et al., 2002). Moreover, Darshan and Manonmani (2015) reviewed that Cu-promoted strand cleavage by prodigiosin (CuProd) induced apoptosis of cancer cells, since they presented higher concentration of Cu(II) than normal cells. Taking into account that the copper concentration available in the ecological niche (Supplementary Table 2) of *Vibrio* sp. might fulfill the level required, it can be hypothesized that the double-strand cleavage of DNA by prodigiosin would be an additional mechanism to survive and compete in a diverse microbial community. In addition, the mode of action of prodigiosin as antimicrobial in *Vibrio* sp. might be also related to its hydrophobicity as has been already reported (Suryawanshi et al., 2015). In that scenario, the knowledge that was obtained about the conditions that promote the prodiginine accumulation by the isolate will be very useful, and more studies would be required to describe in detail the ecological reason of this intriguing characteristic.

The appearance of a red pigmentation was observed only in Site 2 (Supplementary Table 2) of Laguna Tebenquiche in the same sampling campaign when *Vibrio* sp. was isolated. The comparison of the physicochemical parameters of the pigmented and non-pigmented sites at Laguna Tebenquiche, at the same and different campaigns (Supplementary Table 5) evidenced that the chloride concentration, the temperature and the pH of the pigmented brine were in the prodigiosin production range observed and/or reported before (Wei et al., 2005). The occurrence of *Vibrio* in athalasohaline lakes represents another example of phylogenetically close and metabolically similar microorganisms that can be found in rather different environments (Tamames et al., 2016).

The results allow us to conclude that environmental congruence might explain the cellular phenotypes observed in the halo- and psychro-tolerant *Vibrio* sp. (Teb5a1 strain): (i) the environmental conditions where the strain was found agreed well with the strain temperature and salinity tolerance; (ii) the notable temperature range for the growth and the coupling between temperature and salinity tolerance increases the chance of *Vibrio* sp. to survive in an extreme environment with high daily temperature swings encountered at Salar de Atacama (average minimum 2.3°C to average maximum 35°C); (iii) the chloride and temperature dependent production of prodigiosin would be also a mechanism used by *Vibrio* sp. to compete and to survive inside a microbial community in an extreme environment characterized by a high UV radiation as well; (iv) flagellation and motility would further enhance the competitive of *Vibrio* sp. over other members of the microbial community. Finally, considering the diverse biotechnological potential of prodigiosin reported in several microorganisms, like photoprotectant in sunscreens, immunosuppressive activity, antibacterial, antimalarial and anticancerous properties, food colorant, and as colorant for polyolefines (Darshan and Manonmani, 2015), our study of the microbial ecology of this pigment in *Vibrio* sp. can be a first step to explore its diverse biotechnological potential in the future.

## Author Contributions

KG, FR, JC, and CD designed the research experiments; KG and JC conducted the experiments; LE performed the phylogenetic analysis of the isolates; KG and CD analyzed the results and wrote the manuscript.

## Conflict of Interest Statement

The authors declare that the research was conducted in the absence of any commercial or financial relationships that could be construed as a potential conflict of interest.

## Abbreviations

^13^C NMR: carbon-13 nuclear magnetic resonance
^1^H NMR: proton nuclear magnetic resonance
ESI-ITMS: electrospray ionization-ion trap mass spectrometer
LC-MS/MS: liquid chromatography tandem mass spectrometry
TEM: transmission electron microscopy
TLC: thin layer chromatography.
