# Supplementary material for: The Ecological Coherence of Temperature and Salinity Tolerance Interaction and Pigmentation in a Non-marine Vibrio Isolated from Salar de Atacama
Source: Front Microbiol. 2016 Dec 1;7:1943. doi: 10.3389/fmicb.2016.01943 (PMC5130992; doi:10.3389/fmicb.2016.01943)
Supplement: Supplementary file 1 [file Presentation_1.PDF]

Supplementary Material

The ecological coherence of temperature and salinity tolerance interaction and pigmentation in a non-marine vibrio isolated from Salar de Atacama

Karem Gallardo[1], Jonathan Esquivel[1], Francisco Remonsellez[2], Lorena Escudero González[1], Cecilia Demergasso[1]*

*** Correspondence:** Cecilia Demergasso: cdemerga@ucn.cl

Supplemental Table 1. Comparison of physicochemical parameters of ecological niches of several microorganisms from the *Vibrionaceae* family.

	***Vibrio* sp.**	***Vibrio* sp.**	***Vibrio* gazogenes**	***Vibrio* DSM 14379**	***Vibrio* rihizosphaera*e***	***Salinivibrio* costicola**
Primary habitat	Salar de Atacama, Chile	Sea water, India	Salt marshes and marshy areas on the coast of North and South Carolina, USA	Estuarine waters of the Northern Adriatic sea	Rhizosphere region, in India	Marine environment
Temperature range (°C)	4 to 49	15 to 35	25 to 36	10 to 44	20 to 42	5 to 45
optimal temperature (°C)	30	25	26	40	25 to 30	30
Salinity range (%)	0 to 10	4 to 9	0.5 to 12	0.05 to 17	0.1 to 10	0.5 to 12
optimal salinity (%)	2.5	7 to 9	2 to 6	2.5	2	10
pH range	N.D	4 to 9	N.D	5 to 8	N.D	5-10
optimal pH	N.D	7	N.D	7	7	N.D
		(Kirishna et al., 2014)	(Farmer et al., 1988)	(Danevcic, 2014)	(Kumar and Nair, 2007)	(Adams and Russell, 1992; Ventosa et al., 1998)

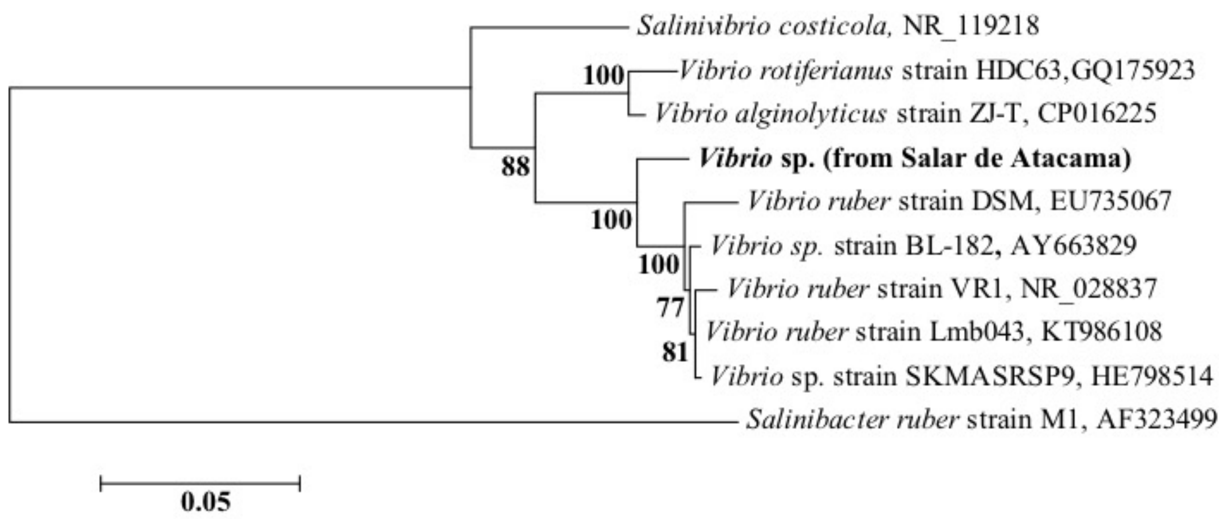

Supplemental Figure 1. Phylogenetic tree highlighting the position of *Vibrio* sp. (bold) isolated from Laguna Tebenquiche, Salar de Atacama, relative to other related type strains within the family *Vibrionaceae*. Sequences were aligned using Clustal and manually corrected. The aligned 16S sequences were used to construct a baseline phylogenetic tree using Neighbor-Joining (NJ), Maximum Parsimony (MP) and Maximum Likelihood (ML) methods. The tree was constructed using the Molecular Evolutionary Genetic Analysis (MEGA) 4 (http://www.megasoftware.net) software with 500 bootstrap replicates.

Supplemental Table 2. Physicochemical parameters in samples from Laguna Tebenquiche, Salar de Atacama*

		**Sampling sites**		
**Parameters**	**Units**	**Teb2 2006**	**Teb2 2007**	**Teb5 2006**
**HCO_3^-**	**mg L^{-1}**	630	616	328
**CO_3^{-2}**	**mg L^{-1}**	155	12	129
**Ca^{2+}**	**mg L^{-1}**	105	134	490
**K^+**	**mg L^{-1}**	1200	479	1200
**Na^+**	**mg L^{-1}**	10100	5086	30500
**SO_4^{-2}**	**mg L^{-1}**	5400	2160	13100
**Cl^-**	**g L^{-1}**	46	7.68	12.9
**pH in situ**		8.16	8.55	7.85
**Conductivity**	**mS**	103.6	18.2	102.5
**Temperature**	**°C**	21.7	18	23.4
		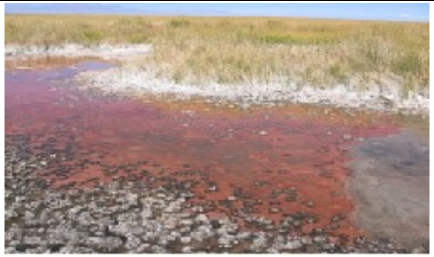	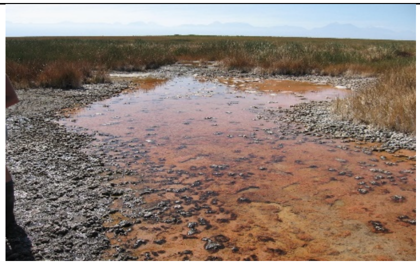	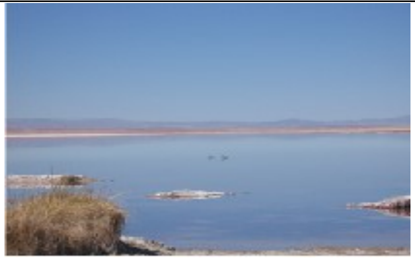

***Sampling Procedure and Measurements:** Sampling expeditions to Laguna Tebenquiche in the Salar de Atacama were in May 2006 and February 2007. For collecting brine and sediment from this place, no permission is required, but there are strict restrictions on sending the samples abroad. The field studies and the experiment did not involve any endangered or protected species. Temperature and pH were measure with a pH meter Orion model 290, the conductivity meter Orion model 115 was used for measuring conductivity in situ. Brine samples were kept in

polyethylene 5-L bottles in an icebox until further processing during the next 24 hr. Brine samples for chemical analysis were filtered through 0.22 μm pore-size (Millipore, Billerica, MA, USA). Major and trace ions in brine samples (Na^+, K^+, Ca^{2+}, SO_4^{2-}, Cl^-, HCO_3^- and CO_3^{2-}) were analyzed by Inductively Coupled Plasma and tritation.

Supplemental Table 3. Methods used to visualize spots under UV/vis light

Method	*Components*	*Proportions*	*Visualization*
**Oleum**	Acetic acid p.a (Merck) 96%	80	Immediate/UV
	Sulfuric acid p.a (Merck) 98%	16	
	Distilled water	4	
**Copper-Phosphoric**	Copper sulphate p.a (Merck)	10% w/v	Immediate
	Phosphoric acid p.a (Merck) 85%	8% v/v	
**Iodine**	Iodine	100%	Immediate/UV
**Nitric-methanol**	Nitric acid p.a (Merck) 65%	2,5% v/v	Immediate/UV
	Methanol p.a (Merck)	97,5% v/v	
**Phosphomolibdic**	Phosphomolibdic acid p.a (Merck)	10% w/v	Immediate
	Ethanol p.a (Merck) 95%	90 mL	

Supplemental Table 4. HPLC parameters for polar and non-polar separation.

Time (min)	Buffer B gradient (%)
**0**	2
**10**	2
**40**	98
**50**	98
**60**	2

Time (min)	Buffer B gradient (%)
Buffer A (polar condition)	0.1% v/v Formic acid
Buffer A (non-polar condition)	10mM ammonium formate
Buffer B (both conditions)	100% Acetonitrile
Flow rate (polar condition)	1 mL/min
Flow rate (non-polar condition)	0.75 mL/min

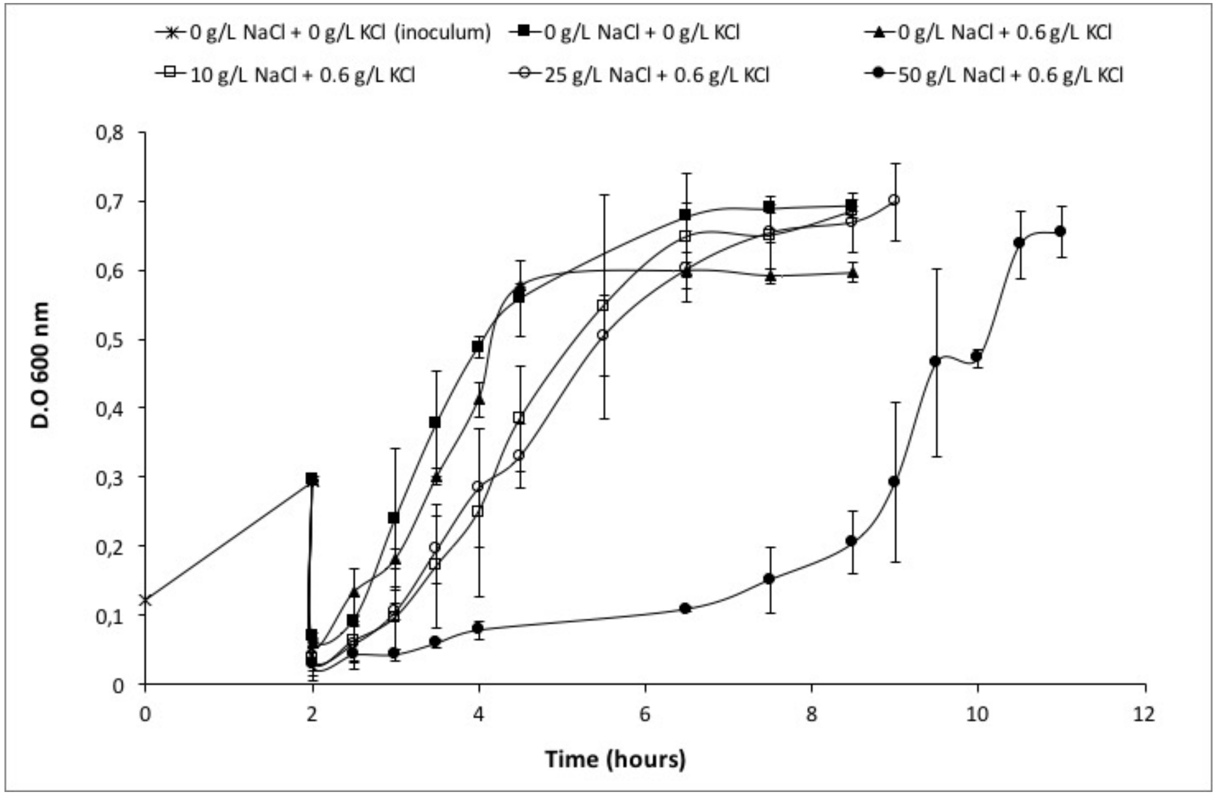

Supplemental Figure 2: Adaptation growth curves of *Vibrio sp* in presence of NaCl (complete). *Vibrio* sp. cells were grown in the absence of NaCl to the early stationary phase (arrow) (✳). Then, cells were transferred to fresh medium containing 0 g/L (■), minimum chloride (▲), 10 g/L (□), 25 g/L (○) and 50 g/L (●).

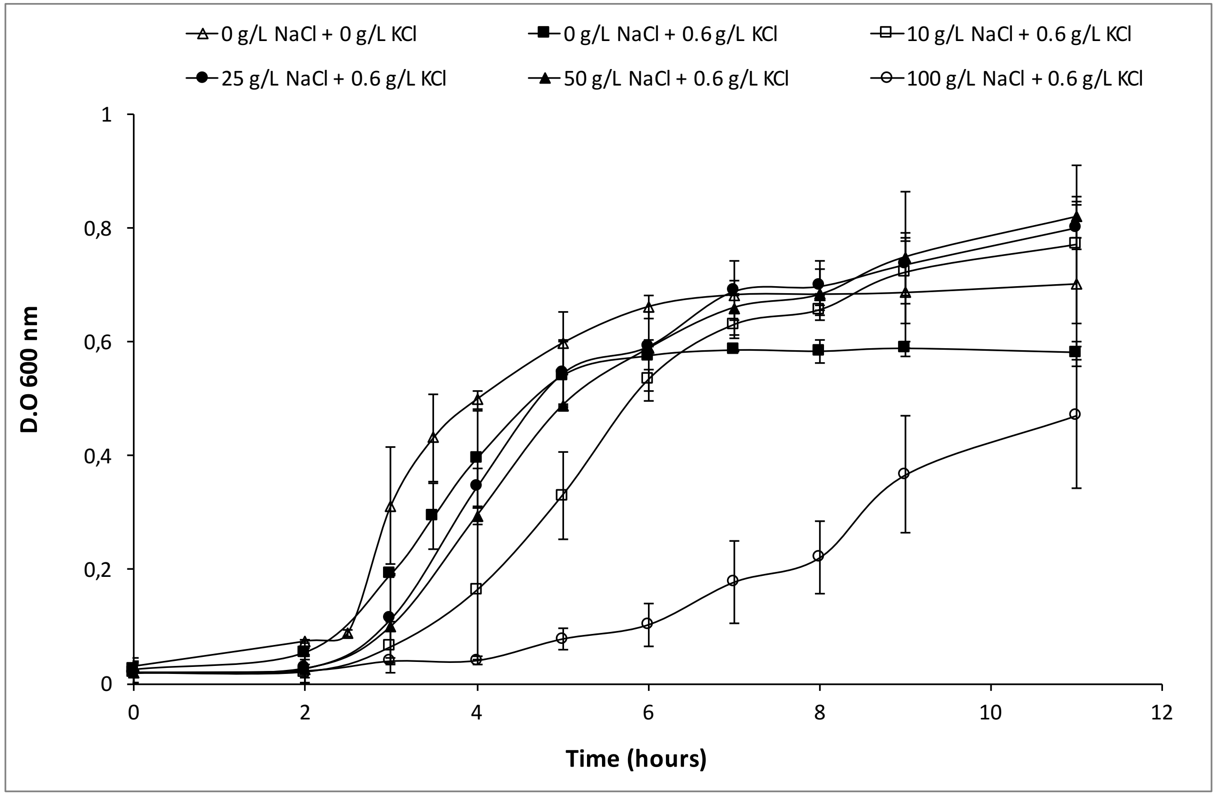

Supplemental Figure 3: Growth curves of *Vibrio sp* in presence of NaCl (complete). *Vibrio* sp. cells were adapted previously to grow in presence of 0 g/L NaCl and KCl (△),0 g/L NaCl and 0.6 g/L KCl (■)(minimum chloride), 10 g/L NaCl (□), 25 g/L NaCl (●), 50 g/L NaCl (▲) and 100 g/L NaCl (○). New cultures were prepared from these cells and used to perform their respective curves.

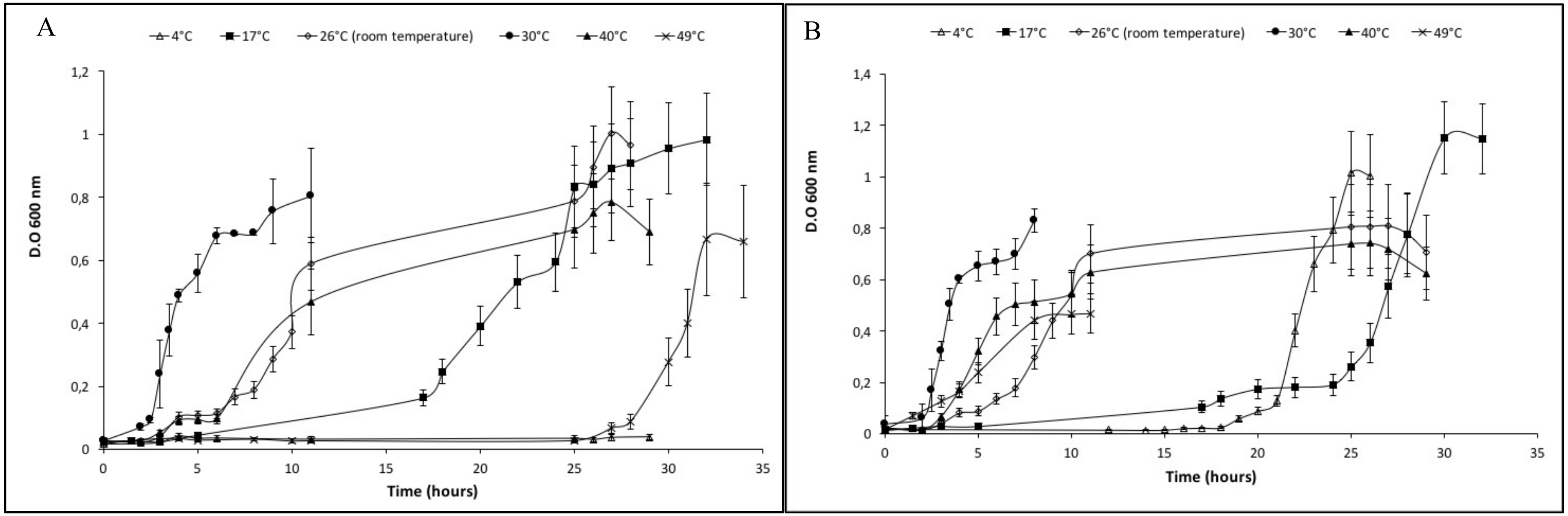

Supplemental Figure 4: Growth curves of *Vibrio* sp. at different temperatures in presence and absence of NaCl (complete). (A) *Vibrio* sp. cells grown in absence of salt (0 g/L NaCl) (B) *Vibrio* sp. cells grown in presence of salt (25 g/L NaCl). The temperature was changed from 4° to 49°C under both growth conditions.

Supplemental Table 5. A) Generation time (T) of *Vibrio* sp. grown in the absence and presence of NaCl at different temperatures. B) Generation time (T) of *Vibrio* sp. grown at 30°C with varied NaCl concentration.

Supplemental Table 5A			Supplemental Table 5B	
Temperature of growth	T at 0 g/L NaCl	T at 25 g/L NaCl	Salt concentration of growth media (growth temperature 30°C)	T at 30°C
4°C	---	3.5	0 g/L NaCl + 0 g/L KCl	2.2
17°C	3.2	3.3	0 g/L NaCl + 0.6 g/L KCl	2.4
26°C	2.7	2.6	10 g/L NaCl + 0.6 g/L KCl	2.7
30°C	2.6	2.4	25 g/L NaCl + 0.6 g/L KCl	2.1
40°C	2.7	2.7	50 g/L NaCl + 0.6 g/L KCl	2.1
49°C	3.9	3.8	100 g/L NaCl + 0.6 g/L KCl	5.2

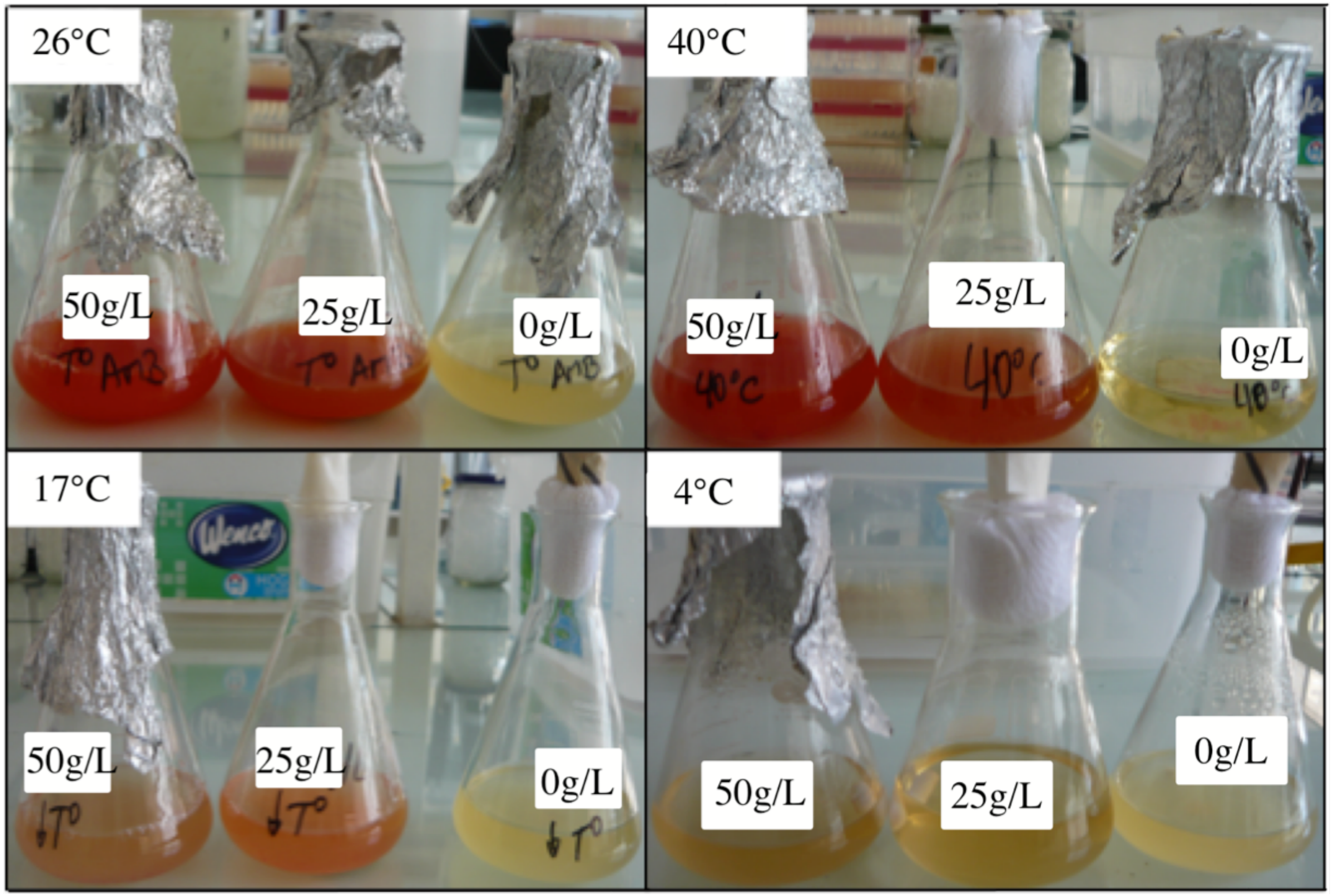

Supplemental Figure 5: Effect of temperature in the pigmentation of *Vibrio* sp.: The microorganism was adapted and grown in presence and absence of NaCl in addition to different temperatures. At low temperatures, the accumulation of pigmentation decreased, whereas at optimal conditions of growth (26 - 40°C) production of prodiginines increased.

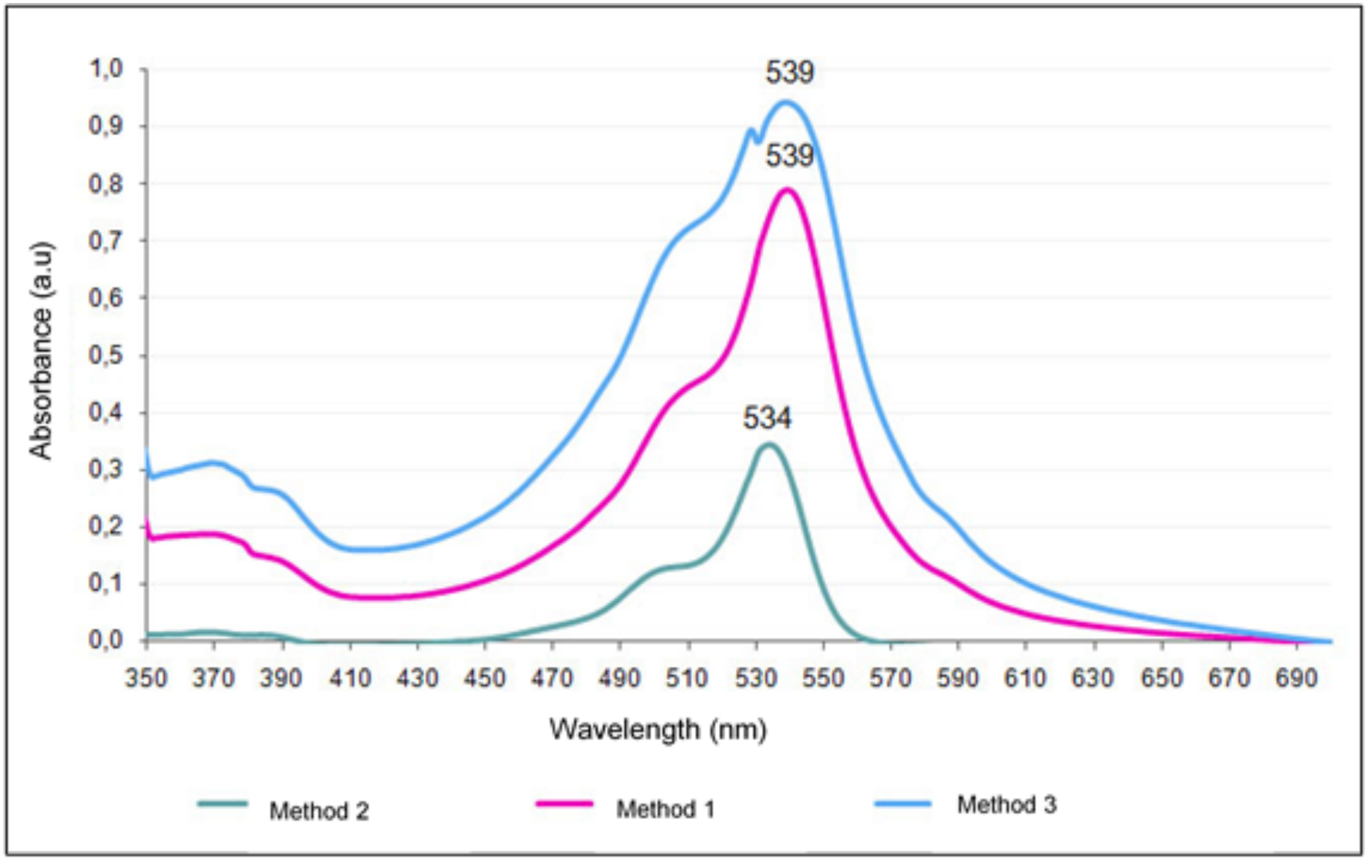

Supplemental Figure 6. UV/Vis spectrum of red pigment extracted by organic solvent. Prodiginines were extracted by three methods based on previous reports (Alihosseini et al., 2008; Cang et al., 2000; Song et al., 2006) with minor modifications as explained in material and methods. The maximal absorbance does not represent quantity.

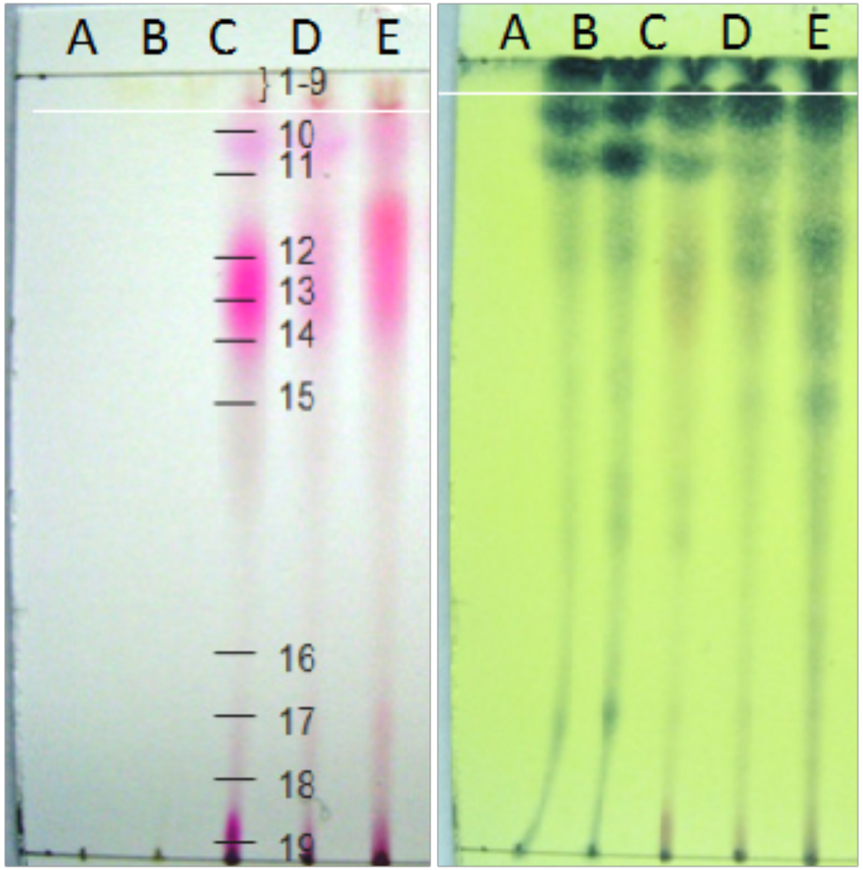

Supplemental Figure 7. Thin layer chromatography of fractions containing prodiginine compounds. Left Figure corresponds to samples loaded on TLC, and right Figure corresponds to spots visualized with phosphomolibdic acid under UV/Vis light. Lane C: first compound mixtures, fractions 336-394. Lane D: second compound mixtures, fractions 395-423. Lane E: third compound mixtures, fractions 424-487. Lanes A and B correspond to fractions containing no prodiginine compounds.

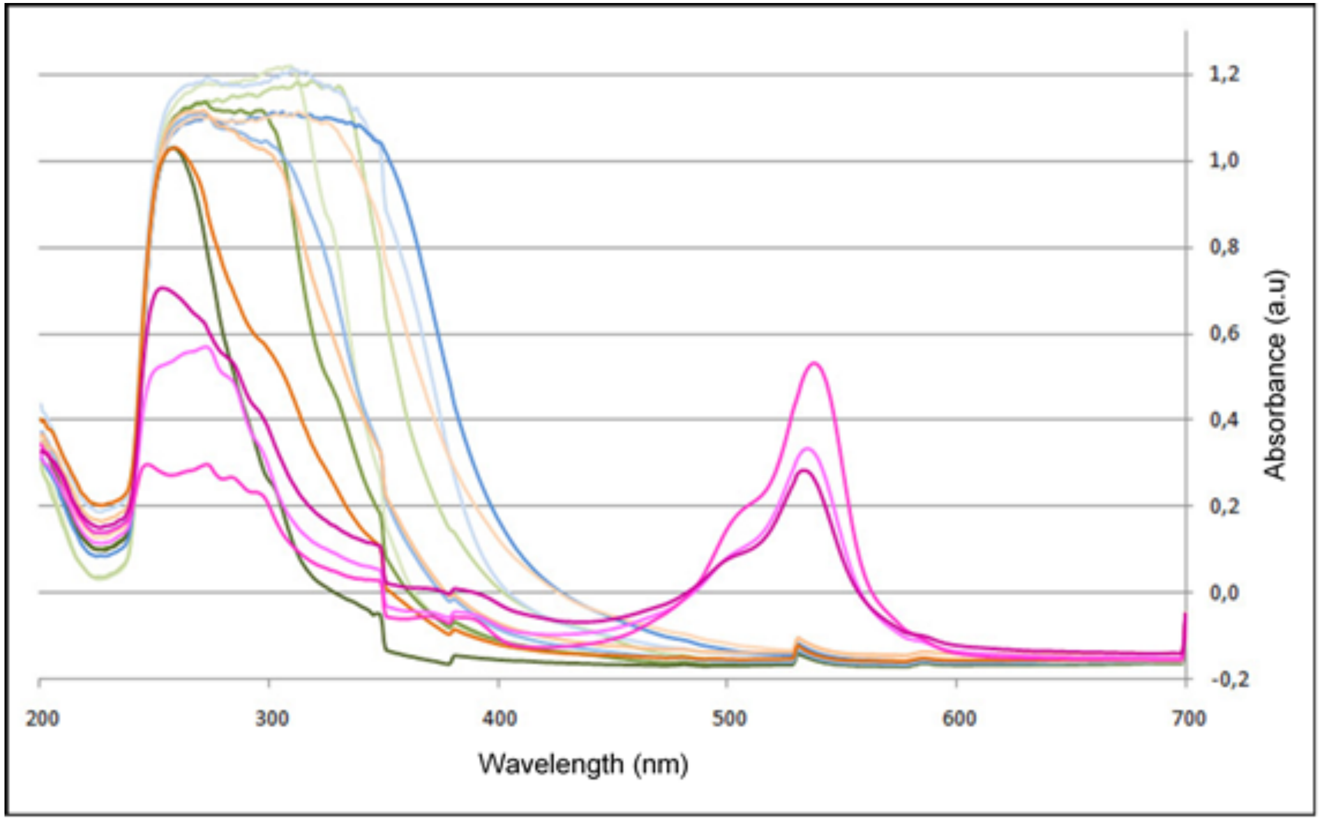

Supplemental Figure 8. UV/Vis spectrum of prodiginine compounds purified from the “first chromatographic column”.

Supplemental Table 6. Identification of prodiginine compounds by LC-MS/MS.

Observed RT (min)	Precursor ion (m/z) $[M+H]^+$	Reported fragments (reference)					Observed fragments m/z (observed intensity)				Compound	Reference of reported MS/MS
**4.0**	296.4	281.0	264.0	252.0			278.8 (93)	263.0 (22)	107.1 (100)	254.1 (43)	2-methyl-3-propylprodiginine	(Kim et al., 2006)
**6.4**	210.9						194.1 (100)	166.0 (45)	100.0 (25)		Prodigiosene	(Kim et al., 2006)
**7.2**	240.2						111.0 (100)	179.0 (94)	153.9 (80)	194.1 (80)	No identified	
**10.7**	436.7						420.5 (32)	405.2 (15)	348.7 (100)	276.7 (93)	Tetradecylprodiginine	(Mo et al., 2008)
**18.8**	324.9	309.2	292.2	252.1	149.0		307.1 (100)	289.1 (5)	251.0 (4)	267.7 (18)	Prodigiosin	(Alihosseini et al., 2008; CHEN et al., 2008; Khanafari et al., 2006; Kim et al., 2006; Liang et al., 2011)
**19.0**	309.7			282.0	240.0	293.0	291.0 (100)	279.0 (18)	249.9 (25)	224.9 (20)	Norprodigiosin	(Kim et al., 2006)
**19.7**	453.4						435.2 (100)	417.3 (7)	336.2 (29)	209.1 (19)	No identified	
**20.7**	394.1	379.3	362.4	252.2	238.2	161.1	377.1 (62)	363.1 (8)	252.0 (6)	238.1 (5)	Undecylprodigiosin	(CHEN et al., 2008; Gerber, 1978; Khanafari et al., 2006; Kim et al., 2006; Mo et al., 2005, 2008)
**41.6**	436.1						330.1 (100)	378.1 (83)	132.9 (40)		No identified	
**43.7**	450.8						420.3 (86)	254.2 (100)	209.7 (40)		No identified	
**54,3**	422.6						406.4 (15)	391.2 (19)	238.9 (100)	211.1 (58)	Methyldodecylprodiginine	(Mo et al., 2005, 2008)
**56.7**	366.3						284.2 (100)				No identified	
**57.5**	365.7						285.2 (100)	195.0 (3)			No identified	
**57.8**	419.9						404.7 (10)	148.9 (100)	275.0 (96)	293.1 (45)	No identified	

**58.0**	338.5	323.0	306.0	252.0			256.2 (100)	283.1 (7)	102.0 (2)		2-methyl-3-hexylprodiginine	(Kim et al., 2006)
**59.1**	392.8	377.2	360.2	252.2	295.2	238.2	375.0 (27)	363.0 (13)	148.9 (100)	281.1 (70)	StreptoRubin B	(CHEN et al., 2008; Gerber, 1978; Khanafari et al., 2006; Mo et al., 2005, 2008)
											Metacycloprodiginine	(Gerber, 1978; Mo et al., 2008)
											Ethylcyclononylprodiginine	(Gerber, 1978; Khanafari et al., 2006)
											Methylcyclodecylprodiginine	(Gerber, 1978; Khanafari et al., 2006)
**61.1**	334.4						151.0 (100)	315.1 (17)	233.1 (15)	288.9 (11)	No identified	
**16.7**	352.2	337.0	320.0	252.0			334.1 (42)	320.0 (4)	253.0 (1)	290.1 (100)	2-methyll-3-hepthylprodiginine	(Kim et al., 2006)
**17.7**	366.4						348.1 (25)	331.2 (1)	304.1 (100)	286.1 (68)	No identified	
**18.9**	380.5						362.1 (38)	349.0 (6)	318.2 (100)	300.1 (69)	No identified	
**20.4**	453.2						435.2 (100)	421.2 (1)	322.2 (64)	209.1 (15)	No identified	
**21.2**	408.6						391.1 (100)	376.1 (4)	328.1 (42)	346.2 (42)	No identified	
**25.6**	351.7						333.1 (11)	305.1 (100)	229.1 (35)	255.1 (13)	No identified	
**29.9**	334.6						318.2 (5)	303.2 (7)	281.1 (13)	315.0 (100)	No identified	
**41.6**	394.5						377.2 (100)	195.0 (9)	243.1 (1)		No identified	
**48.4**	322.0	307.2	290.2	175.1	160.1		306.2 (72)	290.1 (59)	177.1 (15)	291.9 (100)	Cycloprodigiosin	(Alihosseini et al., 2008)
**48.7**	338.7						256.2 (100)	283.0 (10)	102.1 (2)	116.1 (1)	No identified	
**49.1**	364.3						347.2 (24)	332.2 (10)	282.2 (100)	362.1 (53)	No identified	
**49.9**	419.3						402.1 (27)	389.3 (8)	127.0 (100)	148.9 (74)	No identified	

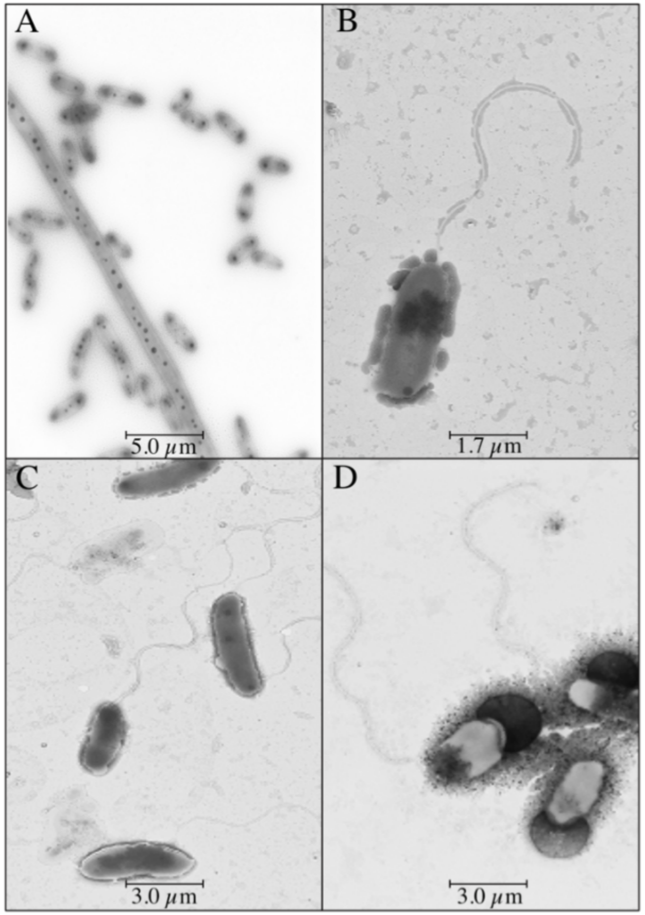

Supplemental Figure 9: NaCl-dependent flagellum in *Vibrio* sp. (complete). Cells were grown in 0 g/L (A), 25 g/L (B), 50 g/L (C), and 100 g/L (D) NaCl, and analyzed by transmission electron microscopy. Arrows indicate the presence of dense granules (A) and flagella (B, C, D).

Supplemental Table 7. Preliminary experiments to verify chloride dependence of *Vibrio* sp. in pigment production and motility.

**Experimental description**	To determine whether motility and pigment production are chloride-dependent, *Vibrio sp*. was grown with different sodium-containing salts. To identify pigment production, the microorganism was grown with 25 g/L NaBr and Na_2SO_4, instead of 25g/L NaCl. For motility assays (Roessler et al., 2000) (swarming and swimming) NaCl, was also exchanged by NaBr and Na_2SO_4. Incubation in both assays (motility and pigment production) was at 30°C in an orbital shaker (100 rpm).
**Results: chloride dependence of *Vibrio sp*. in pigment production.**	As the figure below shows, the microorganism did not show pigmentation under the same physico-chemical growth conditions in the defined medium when NaCl was replaced by 25 g/L of NaBr and Na_2SO_4. This result preliminary showed that the production of the red pigment might be chloride-dependent. However, NaBr and Na_2SO_4 were not equimolar in the growth media to confirm this result.<br>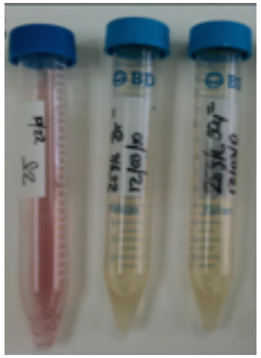<br>NaCl NaBr Na_2SO_4
**Results: chloride dependence of *Vibrio sp*. in motility.**	When cultures were amended with NaBr and Na_2SO_4, instead of NaCl, white colonies and the absence of motility were observed (data not shown). These preliminary results might indicate the fact that the motility is chloride dependent as well. But similar to chloride dependence in pigment production, NaBr and Na_2SO_4 were not equimolar in the growth media to confirm this result.

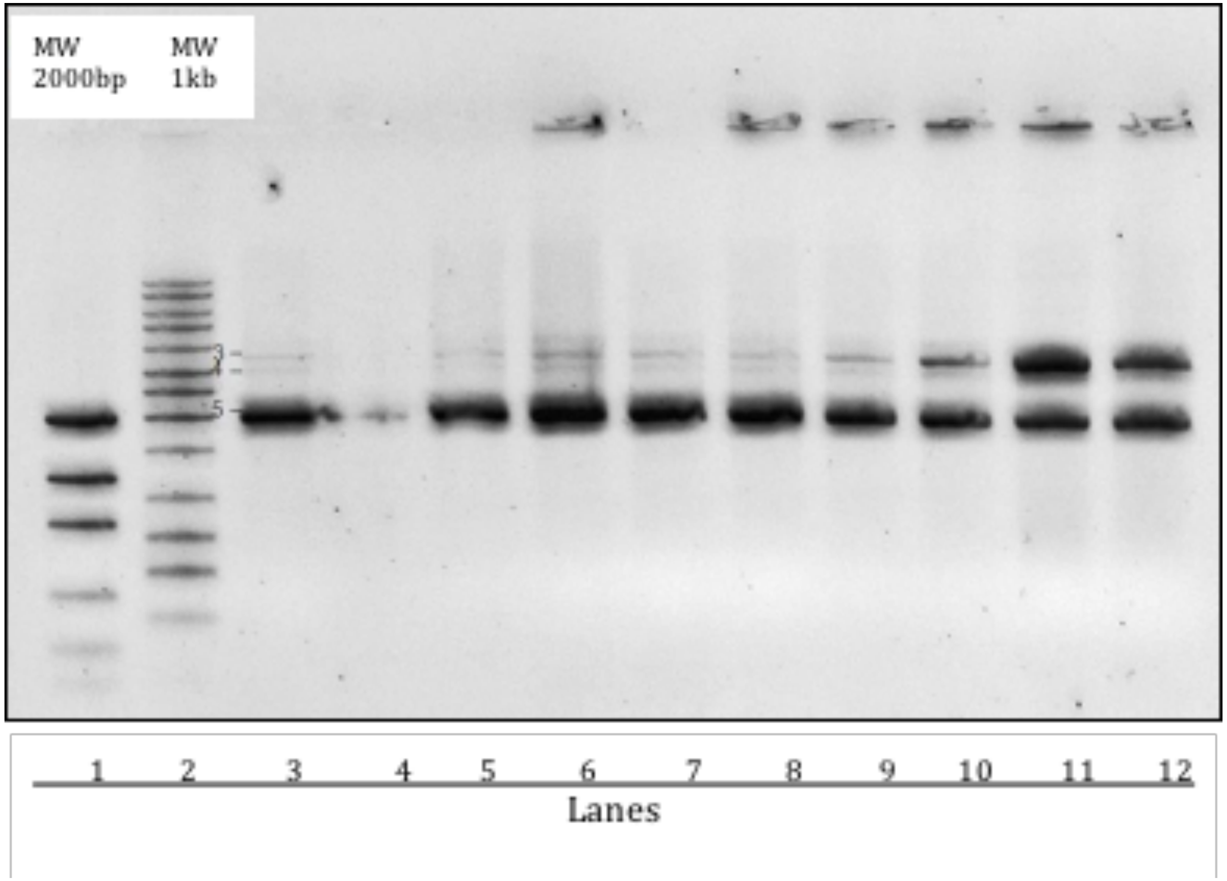

Supplemental Figure 10. Agarose gel electrophoresis of DNA cleavage produced by prodigiosin compounds. Result of agarose gel electrophoresis showing the extent of DNA cleavage produced by the pigment extracted from *Vibrio* sp. in the presence of Cu II, lanes 8-12, after an incubation time of 0 to 120 min at 37 °C. Lane 1 and 2: DNA markers; lane 3: DNA alone; lane 5: DNA in the absence of the bacterial pigment and Cu II; lane 6: DNA + extracted pigment; lane 7: DNA + Cu II. And lane 4: extracted pigment + Cu II.

Supplemental references

Adams, R. L., and Russell, N. J. (1992). Interactive Effects of Salt Concentration and Temperature on Growth and Lipid Composition in the Moderately Halophilic Bacterium Vibrio costicola. *Can J Microbiol* 38, 823–827.

Alihosseini, F., Ju, K.-S., Lango, J., Hammock, B. D., and Sun, G. (2008). Antibacterial Colorants: Characterization of Prodiginines and Their Applications on Textile Materials. *Biotechnol. Prog.* 24, 742–747. doi:10.1021/bp070481r.

Cang, S., Sanada, M., Johdo, O., Ohta, S., Nagamatsu, Y., and Yoshimoto, A. (2000). High production of prodigiosin by Serratia marcescens grown on ethanol. *Biotechnol. Lett.* 22, 1761–1765. doi:10.1023/A:1005646102723.

CHEN, K., RANNULU, N., CAI, Y., LANE, P., LIEBL, A., REES, B., et al. (2008). Unusual Odd-Electron Fragments from Even-Electron Protonated Prodiginine Precursors Using Positive-Ion Electrospray Tandem Mass Spectrometry. *J. Am. Soc. Mass Spectrom.* 19, 1856–1866. doi:10.1016/j.jasms.2008.08.002.

Danevcic, B. and S. (2014). Microbial Ecophysiology of Vibrio ruber. *Food Technol. Biotechnol* 9862, 198–203.

Farmer, J. J., Hickman-Brenner, F. W., Fanning, G. R., Gordon, C. M., and Brenner, D. J. (1988). Characterization of Vibrio metschnikovii and Vibrio gazogenes by DNA-DNA hybridization and phenotype. *J. Clin. Microbiol.* 26, 1993–2000. Available at: http://www.ncbi.nlm.nih.gov/pubmed/3182990.

Gerber, N. N. (1978). Biosynthesis of prodiginines. 13C resonance assignments and enrichment patterns in nonyl-, cyclononyl-, methylcyclodecyl-, and butylcycloheptylprodiginine produced by actinomycete cultures supplemented with 13C-labeled acetate and 15N-labeled nitrate. *Can. J. Chem.* 56, 1155–1163.

Khanafari, A., Assadi, M. M., and Fakhr, F. A. (2006). Review of Prodigiosin, Pigmentation in Serratia marcescens. *Online J. Biol. Sci.* 6, 1–13. doi:10.3844/ojbsci.2006.1.13.

Kim, D., Lee, J. S., Park, Y. K., Kim, J. F., Jeong, H., Oh, T.-K., et al. (2006). Biosynthesis of antibiotic prodiginines in the marine bacterium Hahella chejuensis KCTC 2396. *J. Appl. Microbiol.* 102, 061120055200044–??? doi:10.1111/j.1365-2672.2006.03172.x.

Kirishna, S., Kumar, B. S., Singaracharya, M. A., and Prakasham, R. S. (2014). Process Optimization of Red Pigment production from Vibrio sp Isolated from Marine Source. 2, 79–85.

Kumar, N. R., and Nair, S. (2007). Vibrio rhizosphaerae sp. nov., a red-pigmented bacterium that antagonizes phytopathogenic bacteria. *Int. J. Syst. Evol. Microbiol.* 57, 2241–2246. doi:10.1099/ijs.0.65017-0.

Liang, F.-S., Ho, W. Q., and Crabtree, G. R. (2011). Engineering the ABA Plant Stress Pathway for Regulation of Induced Proximity. *Sci. Signal.* 4, rs2-rs2. doi:10.1126/scisignal.2001449.

Mo, S., Kim, B. S., and Reynolds, K. A. (2005). Production of Branched-Chain Alkylprodiginines in S. coelicolor by Replacement of the 3-Ketoacyl ACP Synthase III Initiation Enzyme, RedP. *Chem. Biol.* 12, 191–200. doi:10.1016/j.chembiol.2004.11.006.

Mo, S., Sydor, P. K., Corre, C., Alhamadsheh, M. M., Stanley, A. E., Haynes, S. W., et al. (2008). Elucidation of the Streptomyces coelicolor Pathway to 2-Undecylpyrrole, a Key Intermediate in Undecylprodiginine and Streptorubin B Biosynthesis. *Chem. Biol.* 15, 137–148. doi:10.1016/j.chembiol.2007.11.015.

Roessler, M., Wanner, G., and Müller, V. (2000). Motility and flagellum synthesis in Halobacillus halophilus are chloride dependent. *J. Bacteriol.* 182, 532–5. doi:10.1128/JB.182.2.532-535.2000.

Song, M.-J., Bae, J., Lee, D.-S., Kim, C.-H., Kim, J.-S., Kim, S.-W., et al. (2006). Purification and characterization of prodigiosin produced by integrated bioreactor from Serratia sp. KH-95. *J. Biosci. Bioeng.* 101, 157–161. doi:10.1263/jbb.101.157.

Ventosa, A., Nieto, J. J., and Oren, A. (1998). Biology of moderately halophilic aerobic bacteria. *Microbiol. Mol. Biol. Rev.* 62, 504–44. Available at: http://www.ncbi.nlm.nih.gov/pubmed/9618450.
